# CSF Biomarkers in COVID-19 Associated Encephalopathy and Encephalitis Predict Long-Term Outcome

**DOI:** 10.3389/fimmu.2022.866153

**Published:** 2022-04-11

**Authors:** Mar Guasp, Guillermo Muñoz-Sánchez, Eugenia Martínez-Hernández, Daniel Santana, Álvaro Carbayo, Laura Naranjo, Uma Bolós, Mario Framil, Albert Saiz, Mircea Balasa, Raquel Ruiz-García, Raquel Sánchez-Valle, The Barcelona Neuro-COVID Study Group

**Affiliations:** ^1^ Neuroimmunology Program, Institut d’Investigacions Biomèdiques August Pi i Sunyer (IDIBAPS), Barcelona, Spain; ^2^ Neurology Service, Hospital Clinic de Barcelona, Barcelona, Spain; ^3^ Centro de Investigación Biomédica en Red, Enfermedades Raras (CIBERER), Madrid, Spain; ^4^ Immunology Department, Centre Diagnòstic Biomèdic, Hospital Clínic, Barcelona, Spain; ^5^ Department of Immunology, Hospital Universitari de Bellvitge, L’Hospitalet de Llobregat, Barcelona, Spain; ^6^ Alzheimer’s Disease and Other Cognitive Disorders Unit, Hospital Clínic de Barcelona, Institut d’Investigacions Biomèdiques August Pi i Sunyer (IDIBAPS), University of Barcelona, Barcelona, Spain

**Keywords:** COVID-19, encephalitis, neurofilaments, neuronal antibodies, SARS-CoV-2, neuro-COVID, encephalopathy, inflammatory cytokines

## Abstract

Patients with coronavirus disease 2019 (COVID-19) frequently develop acute encephalopathy and encephalitis, but whether these complications are the result from viral-induced cytokine storm syndrome or anti-neural autoimmunity is still unclear. In this study, we aimed to evaluate the diagnostic and prognostic role of CSF and serum biomarkers of inflammation (a wide array of cytokines, antibodies against neural antigens, and IgG oligoclonal bands), and neuroaxonal damage (14-3-3 protein and neurofilament light [NfL]) in patients with acute COVID-19 and associated neurologic manifestations (neuro-COVID). We prospectively included 60 hospitalized neuro-COVID patients, 25 (42%) of them with encephalopathy and 14 (23%) with encephalitis, and followed them for 18 months. We found that, compared to healthy controls (HC), neuro-COVID patients presented elevated levels of IL-18, IL-6, and IL-8 in both serum and CSF. MCP1 was elevated only in CSF, while IL-10, IL-1RA, IP-10, MIG and NfL were increased only in serum. Patients with COVID-associated encephalitis or encephalopathy had distinct serum and CSF cytokine profiles compared with HC, but no differences were found when both clinical groups were compared to each other. Antibodies against neural antigens were negative in both groups. While the levels of neuroaxonal damage markers, 14-3-3 and NfL, and the proinflammatory cytokines IL-18, IL-1RA and IL-8 significantly associated with acute COVID-19 severity, only the levels of 14-3-3 and NfL in CSF significantly correlated with the degree of neurologic disability in the daily activities at 18 months follow-up. Thus, the inflammatory process promoted by SARS-CoV-2 infection might include blood-brain barrier disruption in patients with neurological involvement. In conclusion, the fact that the levels of pro-inflammatory cytokines do not predict the long-term functional outcome suggests that the prognosis is more related to neuronal damage than to the acute neuroinflammatory process.

## Introduction

The frequency and spectrum of neurologic manifestations of severe acute respiratory syndrome coronavirus 2 (SARS-CoV-2) infection have been described, but many questions remain unsolved regarding the underlying pathogenic mechanisms related to central nervous system (CNS) involvement (1). Patients with severe coronavirus disease 2019 (COVID-19) develop a syndrome that overlaps with encephalopathy of the critically ill patient at a higher frequency than expected (2, 3). Less frequently, patients develop an acute encephalitis (4). It is unclear if these neurologic syndromes are due to direct infection of the CNS by SARS-CoV-2 given that demonstration of the virus in the brain or cerebrospinal fluid (CSF) has been inconsistent (5). It is possible that these complications are secondary effects resulting from viral-induced mechanisms yet to be elucidated. It has been suggested that CNS disorders associated with COVID-19 may be the result of a cytokine storm syndrome. It is known that the viral infection activates inflammatory, prothrombotic and endothelial pathways that can lead to systemic inflammatory response syndrome (SIRS), in which cytokines are released and may affect the brain function (6, 7). There have been a few observational reports looking at levels of proinflammatory cytokines in patients with both central and peripheral nervous system complications associated to COVID-19. However, sample sizes were small, control groups were missing, CSF analyses were often lacking and follow up was limited, resulting in heterogeneous findings of unclear practical implications. In addition, it is unknown whether cytokine changes might reflect or have predictive or prognostic value. Another possibility could be that SARS-CoV-2 is a trigger of brain autoimmunity, as has been described for other viruses such as herpes simplex virus unleashing autoimmune encephalitis (8).

In this study, we aimed to characterize serum and CSF biomarkers of inflammation (cytokines, antibodies against neural antigens and IgG oligoclonal bands) and neuroaxonal damage (14-3-3 protein and neurofilament light-NfL) in patients with COVID-19 and acute neurologic conditions (neuro-COVID). We compared the profiles of these biomarkers with healthy controls, and among COVID-19 patients with different phenotypes, including those who developed encephalitis, encephalopathy or no CNS complications. We also evaluated the correlation of these biomarkers with the severity of the process at the time of acute COVID-19 and with patients’ long-term functional outcome. We hypothesized that distinct profiles of systemic and intrathecal proinflammatory cytokines and markers of neuroaxonal damage would help to differentiate phenotypes and assess severity in order to select a more appropriate therapeutic strategy, as well as to predict long-term neurologic disability.

## Materials and Methods

### Patients and Controls

All patients with neurologic manifestations associated with COVID-19 whose serum and/or CSF were examined at the Hospital Clínic of Barcelona, Spain, between March 2020 and August 2020, were considered candidates for this study. All cases had definite COVID-19 as confirmed by positive SARS-CoV-2 RNA quantitative reverse transcription polymerase chain reaction (RT-PCR) test on nasopharyngeal swab. We included consecutive patients who provided informed consent for the use of clinical data and sample leftovers for research purposes. Serum and CSF were obtained during the acute stage of COVID-19 for diagnostic purposes as part of standard clinical care.

Clinical information regarding sex, age, past medical history, premorbid functional status (rated with the modified Rankin scale [mRS]) (9), presence or absence of neurological symptoms and signs (**Supplemental Table 1**), intensive care unit (ICU) admission, COVID-19 severity (according to the respiratory status: (a) mild: non- or mild pneumonia or systemic disease, and without supplementary oxygen requirements, (b) moderate: hypoxemia requiring non-invasive supplementary oxygen, and (c) severe: critically ill patients in respiratory failure requiring assisted ventilation, septic shock and/or multi-organ dysfunction), treatments during the acute phase and diagnoses at hospital discharge was obtained by the authors or referring physicians through a structured written questionnaire.

Brain magnetic resonance imaging (MRI), electroencephalogram (EEG) and routine CSF findings were also registered. In all cases, a wide microbiological screening with PCR array and cultures ruled out other infectious diseases in both serum and CSF. Functional outcome at 18 months of follow-up from neurologic symptom onset was assessed with the mRS based on a telephone interview with patients or caregivers conducted by the authors.

For this study, encephalopathy was defined as diffuse brain dysfunction including decreased level of consciousness, cognitive impairment and/or behavioral alterations without signs of acute CNS inflammation, such as CSF pleocytosis and/or brain MRI changes. A diagnosis of encephalitis was given if the patient had decreased level of consciousness, cognitive impairment, behavioral alterations and/or focal abnormalities, along with CSF pleocytosis, inflammatory brain MRI changes and/or new epileptic activity.

Forty-six serum samples from age-matched healthy subjects and 24 CSF samples from age-matched subjects with mild subjective cognitive complaints recruited and followed-up in our institution served as healthy controls (HC). They had objective cognitive performance within the normal range in all tests from a neuropsychological battery, no significant psychiatric symptoms or previous neurologic disease, and normal CSF core Alzheimer’s disease biomarkers, taking as reference the cut-off values described by Antonell et al. (10).

### Laboratory Studies

Fraktalkine (CX3CL1), granulocyte colony stimulating factor (G-CSF), interferon γ (IFN-γ), IFNα2, interleukin (IL)-1β, IL-10, IL-17a, IL-18, IL-1 receptor antagonist (IL-1RA), IL-6, IL-8 (CXCL8), IFN-γ–induced protein 10 (IP-10/CXCL10), macrophage chemoattractant protein 1 (MCP-1/CCL2), MCP-3, monokine induced by interferon (IFN)-gamma (MIG/CXCL9) and tumor necrosis factor-α (TNF-α) levels were quantified in both serum and CSF with a Milliplex^®^ (Merck KGaA, Darmstadt, Germany) custom MAP Human Cytokine/Chemokine/Growth panel and analyzed with LUMINEX^®^ xMAP100. Both serum and CSF NfL concentrations were determined with Simple Plex™ Cartridge Kit containing NfL (ProteinSimple, CA, USA) on Ella™ instrument, according to the manufacturers’ instructions. 14-3-3 protein in CSF was measured by Circulex 14-3-3 Gamma Elisa Kit, MBL. IgG oligoclonal bands were detected by isoelectrofocusing and immunoblotting by Interlab CSF Isoelectrofocusing Kit. Intrathecal IgG synthesis was determined with the IgG/Albumin Ratio by nephelometry in serum and CSF. The following neuronal surface and synaptic antibodies were measured: AMPAR, amphiphysin, CASPR2, DNER, DPPX, GABA_A_R, GABA_B_R, GluK2, GlyR, IgLON5, LGI1, mGluR1, mGluR2, mGluR3, mGluR5, neurexin 3α, and NMDAR. The following onconeural/intracellular and glial antibodies were determined: CV2, GAD, Hu, Ma1/Ma2, Ri, SOX1, Yo, AQP4, GFAP and MOG. The detection of neural antibodies (against neuronal surface antigens and onconeural antigens) was performed by tissue immunohistochemistry, as previously described (11). Samples that produced a neuropil or intraneuronal immunostaining on rat brain immunohistochemistry were subsequently examined with Indirect Immunofluorescence assay (IIFA) or Immunoblot, respectively, as previously described (12). CSF samples of patients with encephalitis were tested for SARS-CoV-2 by RT-PCR.

### Statistical Analyses

All data are described as median and IQR (25th, 75th percentiles), or absolute frequency and percentage for quantitative and qualitative variables, respectively. Serum and CSF biomarkers’ levels were non-normally distributed as tested by the Shapiro-Wilk normality test. Comparisons between clinically defined groups for all biomarkers were performed using Mann-Whitney U test and Kruskal-Wallis test (*post-hoc* analyses between groups were carried out with Bonferroni correction for multiple comparisons). Correlations between serum/CSF biomarkers and the clinical outcome (measured by mRS) at 18 months of follow-up were analyzed by Spearman’s correlation coefficient. Relative cytokine expression between groups was evaluated by principal component analysis and heatmaps. All analyses were addressed considering a two-sided type I error of 5% (p-value <0.05), using SPSS (version 26; IBM Corp, Armonk, NY) and Prism (version 7; GraphPad Software, La Jolla, CA).

### Standard Protocol Approvals, Registrations and Patient Consents

The Ethics’ Committee of Hospital Clínic de Barcelona approved the study. All patients or proxies gave written informed consent for the storage and use of serum and/or CSF leftovers and clinical information for research purposes.

## Results

Clinical and demographic data of the 60 COVID-19 patients included in the study are summarized in **Table 1**. The median age of the patients was 66 years (range, 26-75 years), and 24 (40%) were women. All had new-onset neurological signs or symptoms within 30 days of the first respiratory or systemic COVID-19 symptoms, and required hospital admission. After comprehensive evaluation, patients were classified into the following diagnoses: 1) encephalopathy in 25 (42%) patients (**Supplemental Table 1**); 2) encephalitis in 14 (23%) (**Supplemental Table 1**); 3) peripheral nervous system disorder in 13 (22%, including myopathy in 11 and neuropathy in 2); 4) stroke in 7 (12%), and 5) transverse myelitis in 1 (2%) patient (**Table 1**). Sex and age were equally distributed among the neurologic diagnostic categories. Out of 27 patients who underwent a lumbar puncture, 14 (52%) had normal routine CSF analyses, 10 (37%) had pleocytosis (range 13-95 WBC/µL) and 7 (26%) had elevated protein concentration. Fourteen of 42 (33%) patients with available MRI studies had abnormalities that included multifocal cortical and/or subcortical T2/FLAIR hyperintense lesions in the cerebral hemispheres, basal ganglia and/or brainstem in 3 (7%), mesial temporal T2/FLAIR hyperintense abnormalities in 2 (5%), lesions compatible with stroke in 7 (17%; 5 ischemic and 2 hemorrhagic), and leptomeningeal enhancement and dorsal spinal cord T2/FLAIR hyperintense lesion in one each.

**Table 1:** Demographic and disease characteristics of the 60 patients with COVID-19 and neurologic manifestations.

Female, n (%)	24 (40)
Age, median (IQR)	66 (56-75)
Neurologic diagnostic category, n (%)	
→Encephalopathy	25 (42)
→Encephalitis	14 (23)
→Peripheral nervous system syndrome^1^	13 (22)
→Stroke	7 (12)
→Transverse myelitis	1 (2)
ICU stay, n (%)	27 (45)
Duration of ICU stay, in days, median (IQR)	22 (10-33)
Brain MRI, n (%)	42 (70)
→Normal	28/42 (67)
→Large-vessel ischemic lesion	5/42 (12)
→Multifocal cortical/subcortical T2/FLAIR hyperintense lesions in the cerebral hemispheres, basal ganglia and/or brainstem	3/42 (7)
→Mesial temporal T2/FLAIR hyperintense abnormalities	2/42 (5)
→Intraparenchymal lobar hemorrhagic lesions	2/42 (5)
→Leptomeningeal enhancement	1/42 (2)
→Dorsal spinal cord T2/FLAIR hyperintense lesion	1/42 (2)
Lumbar puncture, n (%)	27 (45)
→Normal	14/27 (52)
→Pleocytosis (>5 WBC/μL), median (range)	10/27 (37), 70 WBC/µL (13-95)
→Increased protein concentration (>60 mg/dL)	7/27 (26)
EEG, n (%)	19 (32)
→Normal	4/19 (21)
→Diffuse slow background activity	11/19 (58)
→Epileptiform activity	4/19 (21)
COVID-19 severity,^2^ n (%)	
→Mild^3^	15 (25)
→Moderate^4^	16 (27)
→Severe^5^	29 (48)
mRS pre-COVID-19, median (IQR)	1 (0-2)
→0-1, n (%)	36 (60)
→2-3, n (%)	15 (25)
→>3, n (%)	9 (15)
mRS at 18 months follow-up, median (IQR)	2 (1-3)
→0-1, n (%)	24/49 (49)
→2-3, n (%)	15/49 (31)
→4-5, n (%)	2/49 (4)
→6, n (%)	8/49 (16)

EEG, electroencephalogram; FLAIR, Fluid-attenuated inversion recovery weighted MRI sequences; ICU, intensive care unit; IQR, interquartile range; MRI, magnetic resonance imaging; mRS, modified Rankin Scale; WBC, white blood cells. (1) Critical care myopathy, 11 (18%); Peripheral nerve syndrome, 2 (3%). (2) According to the respiratory status. (3) Patients with non- or mild pneumonia or systemic disease, and without supplementary oxygen requirements. (4) Patients with hypoxemia requiring non-invasive supplementary oxygen. (5) Critically ill patients in respiratory failure requiring assisted ventilation, septic shock and/or multi-organ dysfunction.

At 18 months of follow-up, the neurological status of the patients was: 24 (49%) with almost complete or totally complete recovery (mRS 0-1), 15 (31%) with mild-moderate neurological disability (mRS 2-3), 2 (4%) with severe functional dependence (mRS 4-5). Eight (16%) patients had died, all due to COVID-related complications; 11 (18%) cases were lost to follow-up.

**Figure 1:**
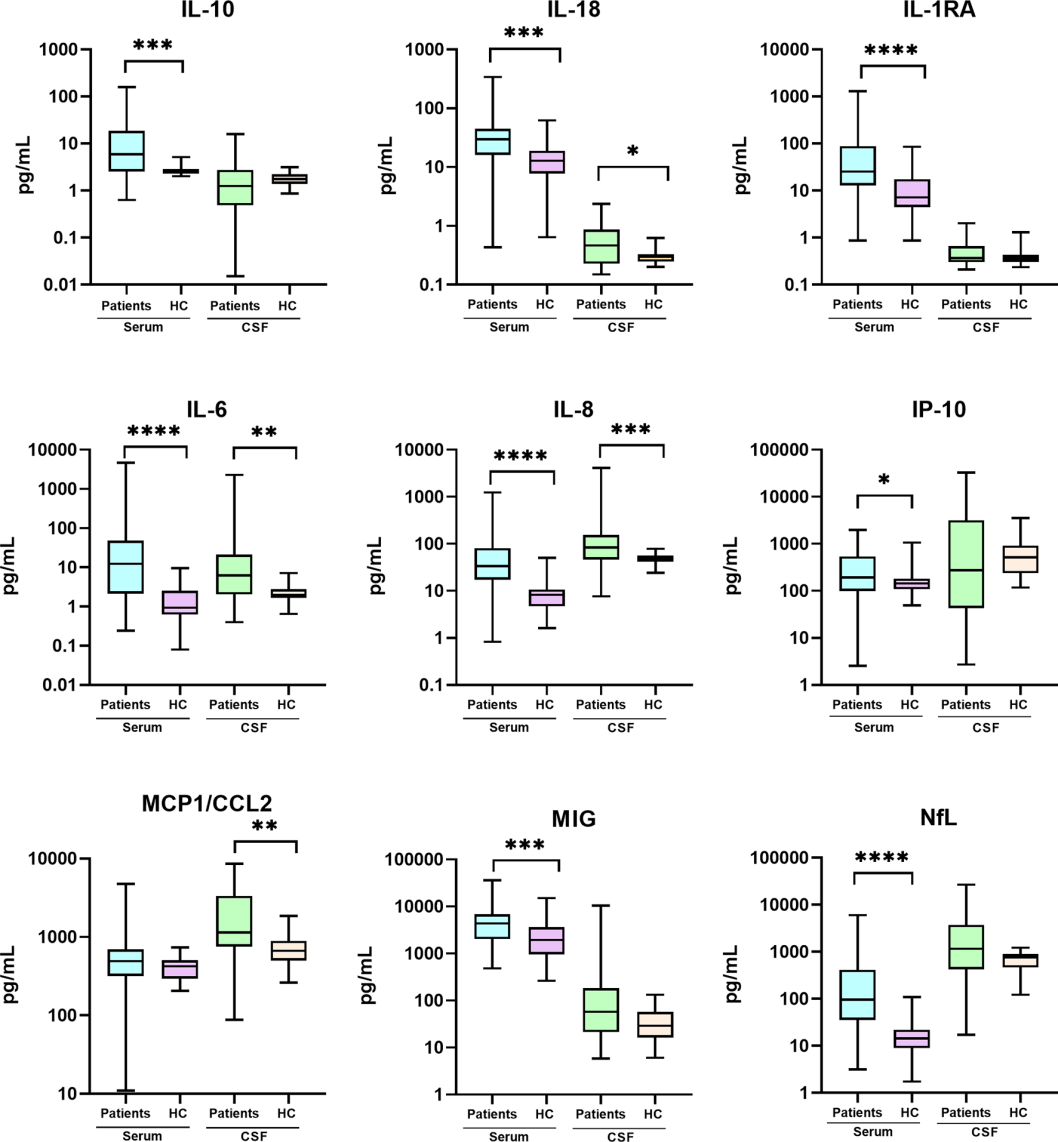
Cytokine and NfL levels in the CSF and serum of patients with COVID-19 and neurological manifestations compared with healthy controls (HC). Comparative analyses were performed using Mann-Whitney U test. CSF, cerebrospinal fluid; IL, interleukin; IP, IFN-γ–induced protein; MCP, macrophage chemoattractant protein; NfL, neurofilaments; MIG, monokine induced by interferon (IFN)-gamma. (*) p<0.05; (**) p<0.01; (***) p<0.001; (****) p<0.0001.

Seventy-five samples from 60 patients were collected and analyzed. Fifteen patients had paired serum/CSF samples, 30 only serum, and 15 only CSF. Eleven of 14 (79%) CSF samples of patients with COVID-19 associated encephalitis were tested for SARS-CoV-2 PCR and all were negative. Compared to HC, the COVID cohort had elevated IL-18, IL-6, and IL-8 levels in both serum and CSF, while IL-10, IL-1RA, IP-10, MIG and NfL were elevated only in serum, and MCP1 only in CSF (**Figure 1**). In contrast, G-CSF, IL-1RA, IL-17a, IL-1b, INFγ, MCP3 and TNFα were undetectable or negligible in the serum and CSF of both the HC and the COVID-19 group. Antibodies against neural antigens (including intracellular, cell-surface, synaptic and glial antibodies) were negative in serum and CSF in all cases.

No differences were found in the serum and CSF levels of cytokines, NfL or 14-3-3 protein when comparing patients according to their neurologic diagnosis as classified in the 5 groups (**Table 2**). Automatic clustering and heatmap analyses based on neurologic diagnoses did not reveal specific biomarker profiles (**Supplemental Figure 1**).

**Table 2:** Serum and CSF biomarkers of inflammation and neuronal damage in 36 patients with COVID-19 associated encephalopathy and encephalitis.

Biomarkermedian (IQR)	COVID-19 encephalopathy (E) (n=25)		COVID-19 encephalitis (e) (n=14)		HC		Significance*
	Serum (n=14)	CSF (n=16)	Serum (n=11)	CSF (n=11)	Serum (n=46)	CSF (n=24)	
OCB	–	0	–	0	–	–	
14-3-3 protein	–	5089 (3133-9847)	–	5272.5 (3176-9049)	–	–	
NfL	241.5 (72.5-857)	1543 (740-2083)	48.4 (4.8-285)	650 (446-4603)	14.4 (9.4-21.9)	764.5 (472.5-896.5)	Serum: **E>HC** (p<0.001) CSF: **E>HC** (p=0.012)
IL-1b	3.7 (1.6-13.9)	0.8 (0.3-0.8)	5.2 (2.3-59.4)	0.8 (0.4-0.8)	1.6 (1.6-8)	0.8 (0.4-0.8)	Serum: **e>HC** (p=0.041)
IL-1RA	34.3 (10.1-76.3)	0.4 (0.3-0.4)	25.8 (5.1-31.1)	0.5 (0.4-0.7)	7.1 (4.5-17.1)	0.4 (0.3-0.4)	Serum: **E>HC** (p<0.001) **e>HC** (p=0.045)
IL-6	16.8 (5.2-61.1)	3.9 (2.1-19.8)	10.2 (1.6-14.9)	11.5 (2.5-38.1)	0.9 (0.6-2.5)	1.9 (1.7-2.8)	Serum: **E>HC** (p<0.001); **e>HC** (p<0.001) CSF: **E>HC** (p=0.019); **e>HC** (p=0.002)
IL-8	34.2 (21-72.2)	83.2 (68.8-161.1)	35.7 (17.6-94.6)	116.3 (45.9-1848)	8.3 (4.7-10.3)	47.3 (41.2-56)	Serum: **E>HC** (p<0.001); **e>HC** (p<0.001) CSF: **E>HC** (p<0.001); **e>HC** (p=0.014)
IL-10	10.1 (2.6-25.2)	1 (0.3-2)	2.8 (2.6-20.4)	1.9 (1.1-8.9)	<2.6*	1.8 (1.4-2.2)	Serum: **E>HC** (p=0.025); **e>HC** (p=0.047) CSF: **E<HC** (p=0.029)
IL-17a	2 (1.3-4.5)	0.6 (0.3-0.6)	1.3 (0.8-20.6)	0.5 (0.2-0.6)	1.3 (1.3-6.1)	0.4 (0.3-0.6)	
IL-18	30.4 (8.5-56.7)	0.5 (0.2-0.7)	26.4 (12.3-88.1)	0.6 (0.2-1)	12.9 (7.8-18.5)	0.3 (0.25-0.3)	CSF: **e>HC** (p=0.026)
IP-10	301.5 (162.4-606.3)	158.7 (32.1-1048.1)	493.7 (60.6-819.5)	612.1 (82.9-27763.5)	142.9 (109.5-178.7)	518.2 (236.4-899.6)	Serum: **E>HC** (p=0.003)
G-CSF	4.8 (4.8-12.3)	<2.4*	14.9 (4.8-34.7)	2.4 (2.4-4.1)	4.8 (4.8-12.3)	<2.4*	
INFα2	8 (8-9.9)	1.5 (0.4-2.1)	8 (8-17.9)	2 (0.3-2.3)	8 (8-33.4)	0.8 (0.5-1.1)	
INFγ	2.3 (1.3-13)	<0.6*	3.3 (1.3-22.2)	<0.6*	4.2 (1.3-17.7)	<0.6*	
TNFα	46.9 (20.1-131.3)	1 (0.9-1.3)	32.3 (23.8-64)	1.7 (0.9-2)	27.7 (16.6-65.2)	1.1 (0.9-1.2)	
Fractalkine	169.6 (111.3-390.7)	51.2 (22.1-75.9)	141.6 (96.5-404)	35 (17.6-72.8)	132.4 (64.3-260.4)	51.3 (38.5-65.6)	
MCP1	622.1 (331.2-892.3)	1216 (873.4-3932.3)	345.4 (285.9-629.7)	1227.2 (613.4-3150.4)	422.6 (297.8-504.3)	666.2 (506.5-882.2)	CSF: **E>HC** (p=0.005); **e>HC** (p=0.023)
MCP3	22.2 (15.4-148.7)	<4*	26.7 (8-81.3)	<4*	19.6 (8-59.6)	<4*	
MIG	4821.5 (2232.1-17391.3)	53.7 (21.3-131.6)	5563.3 (4237.7-9006.4)	68.1 (21.7-692.9)	1936.9 (989.9-3544.9)	29.1 (16.6-55.6)	Serum: **E>HC** (p=0.002); **e>HC** (p=0.002) CSF: **e>HC** (p=0.03)

(*) Only significant differences of pairwise comparisons (between COVID-19 encephalopathy [E], COVID-19 encephalitis [e] and healthy controls [HC]) in post-hoc analyses with Bonferroni correction for multiple comparisons are detailed.

**Figure 2:**
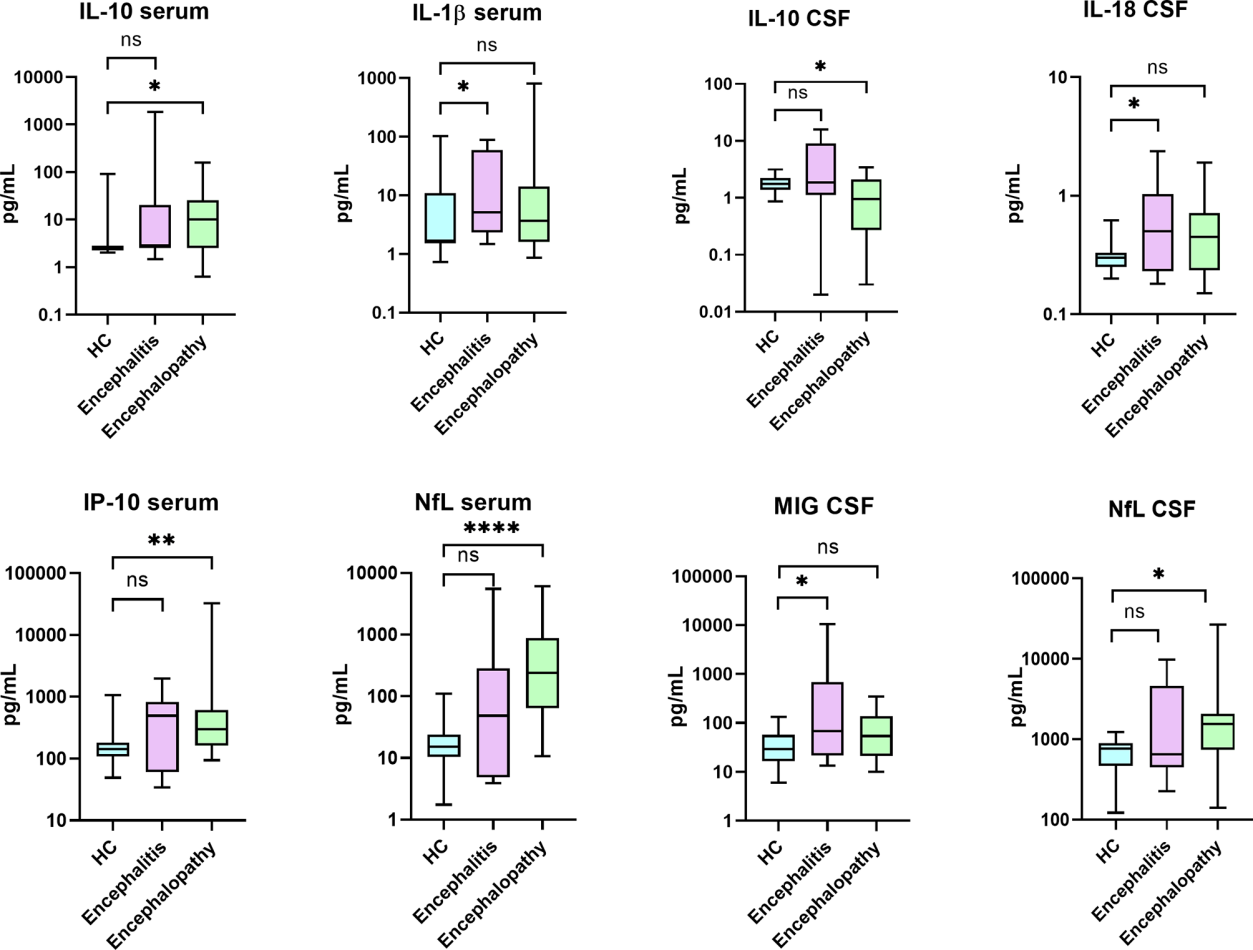
Levels of biomarkers that were found significantly different when comparing patients with COVID-19 associated encephalitis and encephalopathy with healthy controls (HC). Comparative analyses were performed using Mann-Whitney U test. IL, interleukin; IP, IFN-γ–induced protein; NfL, Neurofilaments; MIG, monokine induced by interferon (IFN)-gamma. (*) p<0.05; (**) p<0.01; (****) p<0.0001; ns, not significant.

Compared to HC, patients with encephalitis or encephalopathy presented elevated IL-6 and IL-8 in both serum and CSF, whereas IL-10, IL-1RA, IL-1b and MIG were elevated only in serum, and MCP1 only in CSF (data not shown). However, when specifically compared to HC, patients with encephalopathy had significantly increased levels of IP-10 in serum, and NfL levels in serum and CSF, and lower levels of IL-10 in CSF. In contrast, compared to HC, patients with encephalitis had elevated serum levels of IL-1b and CSF levels of G-CSF, IL-18 and MIG (**Table 2** and **Figure 2**). When we compared patients with inflammatory (encephalitis and myelitis, n=15, 25%) with non-inflammatory (45, 75%) neuro-COVID based on CSF and MRI findings, a significant increase of G-CSF was found in the serum of the patients with inflammatory neuro-COVID (median 17.7 [IQR 4.8-31.7] vs 4.8 [4.8-6.2]; p=0.049).

**Figure 3:**
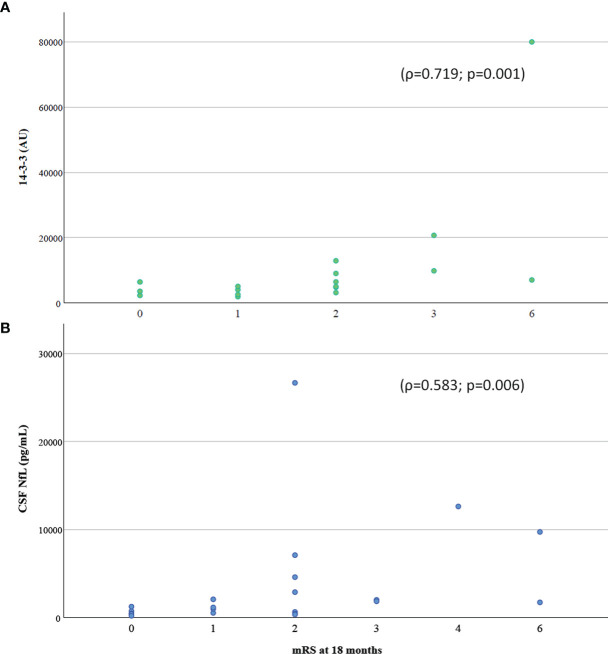
Correlation of **(A)** 14-3-3 protein and **(B)** NfL in CSF with functional outcome assessed with mRS at 18 months follow-up. CSF, cerebrospinal fluid; mRS, modified Rankin Scale.

The severity of the acute COVID-19 systemic disease according to the respiratory status was associated with CSF levels of 14-3-3 (ρ=0.689; p=0.018) and NfL (ρ=0.45; p=0.043), and serum levels of IL-18 (ρ=0.498; p=0.005), IL-1RA (ρ=0.487; p=0.025), IL-8 (ρ=0.367; p=0.014) and serum NfL (ρ=0.677; p<0.001). Finally, the long-term functional outcome, as measured by the mRS, significantly correlated with CSF levels of 14-3-3 protein (ρ=0.719; p=0.001) and CSF NfL (ρ=0.583; p=0.006), but not with serum NfL levels (ρ=0.199; p=0.244) (**Figure 3**).

## Discussion

In this prospective cohort of 60 patients with COVID-19 and associated neurologic manifestations, we confirmed the presence of increased levels of several proinflammatory cytokines in both CSF and serum. This study also showed the association of neuroaxonal damage markers 14-3-3 and NfL, and proinflammatory cytokines IL-18, IL-1RA and IL-8 with COVID-19 severity in the acute phase, and interestingly, the significant association of CSF 14-3-3 and CSF NfL levels with the long-term functional outcome. In contrast, we did not detect the presence of antibodies against neural antigens in any of the patients who developed neurologic manifestations, including those with encephalitis and encephalopathy syndromes.

Previous studies (13–15) analyzing serum and CSF inflammatory markers in COVID-19 patients with neurologic manifestations have shown contradictory results, although most studies reported increased levels of IL-6 in serum and CSF (16), which has been used to justify, in part, the use of tocilizumab in those COVID-19 patients with a more severe inflammatory response. In one study, the demonstration that patients with COVID-19 associated encephalitis and encephalopathy had different serum and CSF individual cytokines levels compared to HC led to the suggestion that these cytokines might be useful for distinguishing between inflammatory neurologic syndromes and encephalopathy (13). However, no direct comparison between the encephalitis and encephalopathy groups was done. In our study, although we also found differences in serum and CSF cytokine profiles when comparing the encephalitis or encephalopathy cohorts with HC, it is important to note that no significant differences were observed in the direct comparison between encephalitis and encephalopathy groups. Thus, our findings do not support the diagnostic utility of measuring cytokines to distinguish between inflammatory CNS syndromes and encephalopathy, and suggest that COVID-19-related encephalopathy likely has a cytokine-mediated inflammatory pathogenesis similarly to what has been observed in other conditions such as CAR-T neurotoxicity or ICANS (17, 18). In contrast to previous reports (13), we detected higher levels of MCP-1/CCL-2 in the CSF of patients with neuro-COVID as compared with HC. Recent evidence has shown that MCP-1/CCL2 is involved in disruption of the blood-brain barrier in the context of cerebral damage, such as intracerebral hemorrhage (19). Therefore, it has emerged as an important chemokine that plays a pivotal role in many CNS disorders, especially those related to -inflammation (20). For example, it has been recently described (21) that MCP-1/CCL2 overexpression worsens tau pathology by an inflammatory response mediated by microglial activation. Our results would support the involvement of MCP-1 in the inflammatory process promoted by SARS-CoV-2 infection in patients with neurological involvement.

Regarding neuronal damage in neuro-COVID-19 patients, we found increased levels of both the neuroaxonal marker NfL and the synaptic marker 14-3-3. Interestingly, unlike previous small cohort studies that focused primarily on the role of NfL as a biomarker of disease severity (16, 22, 23), we have seen that both CSF NfL and 14-3-3 levels significantly correlate with neurological status at 18 months of follow-up. That is, higher basal levels of these neuronal damage markers were correlated with a poorer clinical outcome. In contrast, the functional outcome was not predicted by the levels of pro-inflammatory cytokines, suggesting that long-term functional prognosis is more closely related to neuronal damage rather than the acute neuroinflammatory process, similar to other neuroinmunological diseases (24, 25).

Lastly, the fact that none of the patients harbored antibodies against neural antigens at the onset of neurologic involvement by acute COVID-19, including those patients who presented with inflammatory features of the CSF and/or brain MRI, would suggest that the infection by SARS-CoV-2 is unlikely to trigger an intrathecal B-cell-specific autoimmune response. This could be due to its low neurovirulence and the lack of viral-induced release of neural proteins during the acute CNS dysfunction, making cytokine storm the most likely cause of inflammatory CNS injury. However, we do not know whether a delayed neural autoimmune response may occur in some patients with persisting neurologic symptoms.

There are a number of limitations to this study. Sample size, even though greater than previous studies, is still relatively small and limits the statistical analyses. The study did not include a control group of patients with COVID-19 without neurologic manifestations or a control group with a different viral infection. Another possible selection bias is the enrollment of patients with available serum/CSF samples, and that lumbar punctures for CSF examination were performed based on standard clinical indication (not systematically in all neuro-COVID patients). However, the study included a comprehensive serum and CSF testing at disease onset, and prolonged clinical follow-up. Further longitudinal studies with larger samples sizes are needed to confirm the correlations of CSF 14-3-3 and NfL levels in patients that progress to a long-standing neuro-COVID disease.

In summary, this study provides further evidence of distinct systemic and intrathecal proinflammatory cytokine profiles in patients with acute neurologic manifestations associated with COVID-19, unrelated to neural autoimmunity. We also show that increased CSF levels of markers of neuronal damage during the acute phase of COVID-19 are associated with worse long-term clinical outcome of the patients, supporting their potential use as prognostic markers.

## Data Availability Statement

The original contributions presented in the study are included in the article/**Supplementary Material**. Further inquiries can be directed to the corresponding authors.

## Ethics Statement

The studies involving human participants were reviewed and approved by The Ethics’ Committee of Hospital Clínic de Barcelona. The patients/participants provided their written informed consent to participate in this study.

## Barcelona Neuro-COVID Study Group

Virginia Casado Ruiz (Hospital de Mataró, Consorci Sanitari del Maresme, Spain), Marta Perez Moreno (Hospital General Granollers, Spain), Esther Catena Ruiz (Consorci Sanitari Alt Penedès-Garraf, Spain), Marta Palacín Larroy (Hospital Ernest Lluch de Calatayud, Spain), Nicolau Guanyabens (Hospital de Mataró, Spain) Eloi Giné-Servén (Hospital de Mataró, Spain) Javier Villacieros-Álvarez (Hospital Universitario Rey Juan Carlos, Spain), Natalia Hernando Quintana (Hospital Obispo Polanco de Teruel, Spain), Isabel Pellicer-Espinosa (Hospital Comarcal del Noroeste, Caravanca de la Cruz, Spain), Eloi Giné Servén (Hospital de Mataró, Consorci Sanitari del Maresme, Spain), Nicolau Guanyabens (Hospital de Mataró, Consorci Sanitari del Maresme, Spain), Elba Pascual Goñi (Hospital de la Santa Creu i Sant Pau, Spain), Delon La Puma (Hospital Quironsalud Barcelona, Spain).

## Author Contributions

RS-V and MG designed and conceptualized the study, and obtained funding. RR-G, GM-S, EM-H and AS participated in the study design. MG, GM-S, RS-V, and RR-G interpreted the data and wrote the manuscript. MG, EM-H, DS, AC, AS, RS-V, and the Barcelona Neuro-COVID study group collected and reviewed the clinical data. RR-G, GM-S, MG, LN, UB, and MF laboratory studies and/or statistical analyses. MB (MSc statistics) reviewed the statistical analyses. All authors contributed to the article and approved the submitted version.

## Funding

This study was funded by the Fondo de Mecenazgo COVID-19 from Hospital Clínic de Barcelona, Barcelona, Spain. Dr. M. Guasp is a recipient of a Resident Award “Josep Font,” granted by Hospital Clínic de Barcelona, Research, Innovation and Education Departments.

## Conflict of Interest

The authors declare that the research was conducted in the absence of any commercial or financial relationships that could be construed as a potential conflict of interest.

## Publisher’s Note

All claims expressed in this article are solely those of the authors and do not necessarily represent those of their affiliated organizations, or those of the publisher, the editors and the reviewers. Any product that may be evaluated in this article, or claim that may be made by its manufacturer, is not guaranteed or endorsed by the publisher.

## Supplementary Material

Supplementary Table 1 Clinical features of patients with encephalopathy and encephalitis syndromes associated to COVID-19. EEG, electroencephalogram; ICU, intensive care unit; IQR, interquartile range; MRI, magnetic resonance imaging; mRS, modified Rankin Scale; WBC, white blood cells. **(A)** Two cases compatible with acute disseminated encephalomyelitis (ADEM) and 2 with limbic encephalitis, but none with acute necrotizing encephalopathy (ANE). **(B)** Three with multifocal myoclonus and 1 with akinetic-rigid syndrome. **(C)** Three with multifocal cortical/subcortical T2/FLAIR hyperintense lesions in the cerebral hemispheres, basal ganglia and/or brainstem; 2 with mesial temporal T2/FLAIR hyperintense abnormalities; and 1 with leptomeningeal enhancement. **(D)** One in focal status epilepticus. **(E)** According to the respiratory status, using same criteria as **Table 1**.

Supplementary Figure 1 Hierarchical clustering of patients according to cytokine levels in CSF **(A)** and serum **(B)** using ClustVis (https://biit.cs.ut.ee/clustvis/).
