# Supplementary material for: CSF Biomarkers in COVID-19 Associated Encephalopathy and Encephalitis Predict Long-Term Outcome
Source: Front Immunol. 2022 Apr 11;13:866153. doi: 10.3389/fimmu.2022.866153 (PMC9035899; doi:10.3389/fimmu.2022.866153)
Supplement: Supplementary Table 1 — Clinical features of patients with encephalopathy and encephalitis syndromes associated to COVID-19. EEG, electroencephalogram; ICU, intensive care unit; IQR, interquartile range; MRI, magnetic resonance imaging; mRS, modified Rankin Scale; WBC, white blood cells. (A) Two cases compatible with acute disseminated encephalomyelitis (ADEM) and 2 with limbic encephalitis, but none with acute necrotizing encephalopathy (ANE). (B) Three with multifocal myoclonus and 1 with akinetic-rigid syndrome. (C) Three with multifocal cortical/subcortical T2/FLAIR hyperintense lesions in the cerebral hemispheres, basal ganglia and/or brainstem; 2 with mesial temporal T2/FLAIR hyperintense abnormalities; and 1 with leptomeningeal enhancement. (D) One in focal status epilepticus. (E) According to the respiratory status, using same criteria as Table 1 . [file Table_1.docx]

**Supplementary Table 1**: Clinical features of patients with encephalopathy and encephalitis syndromes associated to COVID-19.

|  | **Encephalopathy (n=25)** | **Encephalitis (n=14)**^a^ |
| --- | --- | --- |
| Female, n (%) | 9 (36) | 7 (50) |
| Age, median (IQR) | 71 (61-78) | 60 (50-65) |
| General neurological findings; n (%)   - Cognitive alterations - Psychiatric alterations - Seizures; with status epilepticus - Altered level of consciousness - Abnormal movements - Focal deficits (motor or sensory) - Brainstem and/or cerebellar signs | 25 (100)  20 (80)  0 (0), 0 (0)  21 (84)  4 (16)^b^  0 (0)  0 (0) | 14 (100)  14 (100)  6 (43), 1 (7)  7 (50)  0 (0)  4 (29)  2 (14) |
| ICU stay, n (%)  Duration of ICU stay, in days, median (IQR) | 15 (60)  31 (15-35) | 5 (36)  16 (10-40) |
| Abnormal brain MRI, n (%) | 0/21 (0) | 6/13 (46)^c^ |
| Abnormal CSF, n (%)   - Pleocytosis (>5 WBC/μL), median [range] - Increased protein concentration (>60 mg/dL), median [range] | 2/16 (13)  0/16 (0)  2/16 (13), 66 mg/dL [66-103] | 11/11 (100)  10/11 (91), 70 WBC/µL (13-95)  5/11 (45), 115 mg/dL [91-220] |
| Abnormal EEG, n (%)   - Diffuse slow background activity - Epileptic activity | 4/7 (57)  4/7 (57)  0 (0) | 11/12 (92)  7/12 (58)  4/12 (33)^d^ |
| COVID-19 severity,^e^ n (%)   - Mild - Moderate - Severe | 3 (12)  7 (28)  15 (60) | 6 (43)  3 (21)  5 (36) |
| mRS pre-COVID-19, median (IQR)   - 0-1, n (%) - 2-3, n (%) - >3, n (%) | 1 (0-2)  15 (60)  10 (40)  0 (0) | 1 (0-1)  9 (64)  4 (29)  1 (7) |
| mRS at 18 months follow-up, median (IQR)   - 0-1, n (%) - 2-3, n (%) - 4-5, n (%) - 6, n (%) | 2 (1-3)  8/21 (38)  10/21 (48)  0/21 (0)  3/21 (14) | 2 (1-2)  7/13 (54)  4/13 (31)  0/13 (0)  2/13 (15) |

EEG: electroencephalogram; ICU: intensive care unit; IQR: interquartile range; MRI: magnetic resonance imaging; mRS: modified Rankin Scale; WBC: white blood cells. (a) Two cases compatible with acute disseminated encephalomyelitis (ADEM) and 2 with limbic encephalitis, but none with acute necrotizing encephalopathy (ANE). (b) Three with multifocal myoclonus and 1 with akinetic-rigid syndrome. (c) Three with multifocal cortical/subcortical T2/FLAIR hyperintense lesions in the cerebral hemispheres, basal ganglia and/or brainstem; 2 with mesial temporal T2/FLAIR hyperintense abnormalities; and 1 with leptomeningeal enhancement. (d) One in focal status epilepticus. (e) According to the respiratory status, using same criteria as Table 1.

**Supplementary Figure 1**: Hierarchical clustering of patients according to cytokine levels in CSF (Supplementary Fig 1A) and serum (Supplementary Fig 1B) using ClustVis (https://biit.cs.ut.ee/clustvis/).
