# Supplementary figures and images for: CSF Biomarkers in COVID-19 Associated Encephalopathy and Encephalitis Predict Long-Term Outcome
Source: Front Immunol. 2022 Apr 11;13:866153. doi: 10.3389/fimmu.2022.866153 (PMC9035899; doi:10.3389/fimmu.2022.866153)

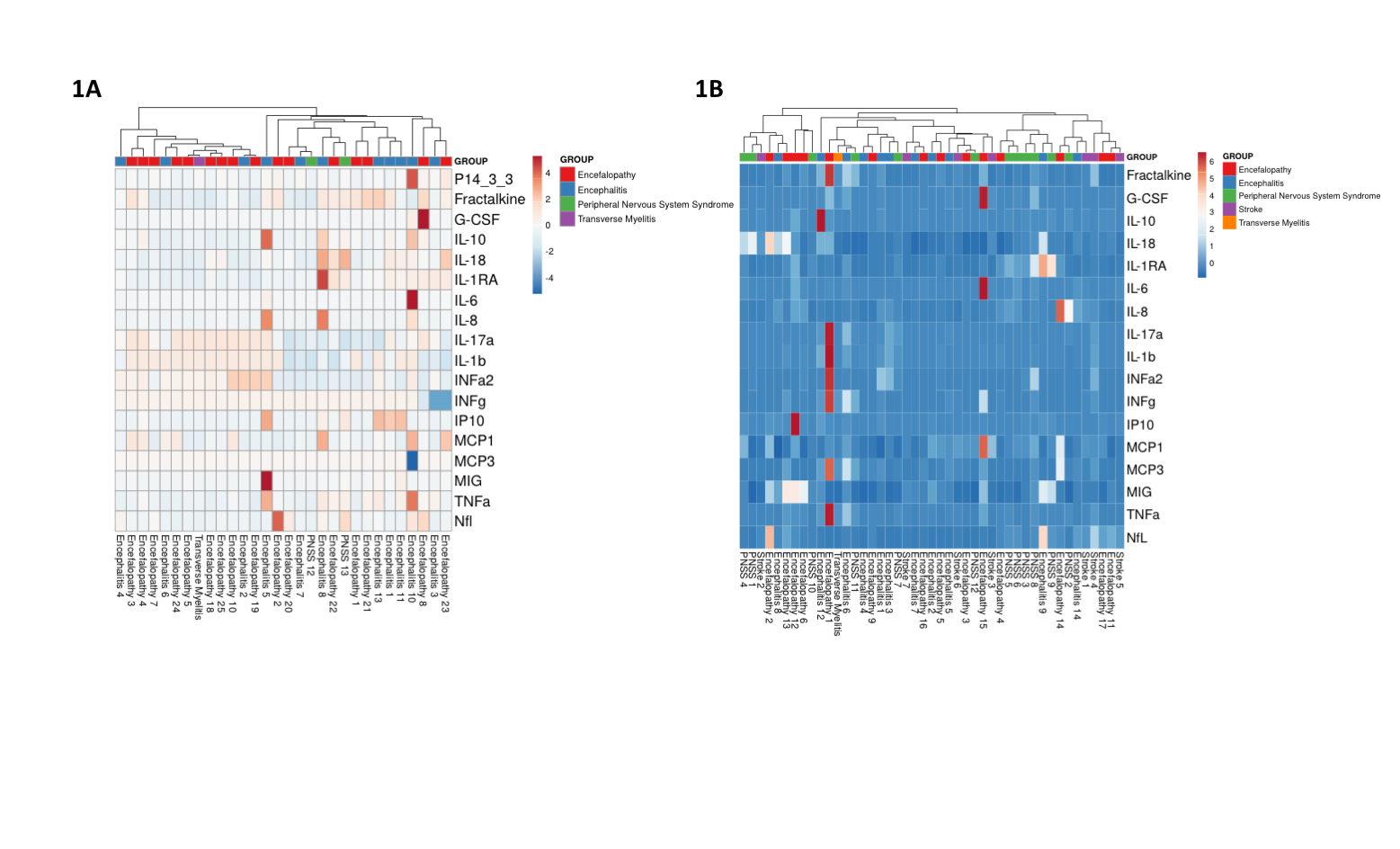

Supplement: Supplementary Figure 1 — Hierarchical clustering of patients according to cytokine levels in CSF (A) and serum (B) using ClustVis (https://biit.cs.ut.ee/clustvis/). [file Image_1.tiff]
